# MiRNA‐501‐3p and MiRNA‐502‐3p: A promising biomarker panel for Alzheimer's disease

**DOI:** 10.1002/ctm2.70389

**Published:** 2025-07-09

**Authors:** Davin Devara, Bhupender Sharma, Gunjan Goyal, Daniela Rodarte, Aditi Kulkarni, Nathan Tinu, Ayana Pai, Subodh Kumar

**Affiliations:** ^1^ Center of Emphasis in Neuroscience Department of Molecular and Translational Medicine Paul L. Foster School of Medicine Texas Tech University Health Sciences Center El Paso El Paso Texas USA; ^2^ L. Frederick Francis Graduate School of Biomedical Sciences Texas Tech University Health Sciences Center El Paso El Paso Texas USA

**Keywords:** Alzheimer's disease, biomarker, CSF, MiR‐501‐3p, MiR‐502‐3p, serum

## Abstract

**Introduction:**

Alzheimer's disease (AD) lacks a less invasive and early detectable biomarker. Here, we investigated the biomarker potential of miR‐501‐3p and miR‐502‐3p using different AD sources.

**Methods:**

MiR‐501‐3p and miR‐502‐3p expressions were evaluated in AD cerebrospinal fluid (CSF) exosomes, serum exosomes, familial and sporadic AD fibroblasts and B‐lymphocytes by qRT‐PCR analysis. Further, miR‐501‐3p and miR‐502‐3p expressions were analysed in APP, Tau plasmid transfected cells media exosomes and in different brain cell types.

**Results:**

MiR‐501‐3p and miR‐502‐3p expressions were significantly up‐regulated in AD CSF exosomes relative to controls. MiRNA levels were high in accordance with amyloid plaque and NFT density in multiple brain regions. Similarly, both miRNAs were elevated in AD and MCI serum exosomes compared with controls. MiR‐502‐3p expression was high in familial AD and sporadic AD B‐lymphocytes. MiR‐501‐3p and miR‐502‐3p expression were elevated intracellularly and secreted extracellularly in response to APP and Tau pathology. Finally, neurons and astrocytes displayed high expression of these miRNAs.

**Discussion:**

These results suggest that miR‐501‐3p and miR‐502‐3p could be promising biomarkers for AD.

**Key points:**

- MiR‐501‐3p and miR‐502‐3p expression is elevated in AD CSF exosomes, AD serum exosomes, AD B‐lymphocytes and Aβ‐ and Tau‐treated cells.
- MiR‐501‐3p and miR‐502‐3p are correlated with amyloid plaque and NFT tangle density in specific brain regions.
- MiR‐501‐3p and miR‐502‐3p are highly expressed in neurons and astrocytes, suggesting that these cells are the source of miRNA secretion.
- MiR‐501‐3p and miR‐502‐3p could be a promising biomarker panel for AD

## Graphical abstract

1. MiR‐501‐3p and miR‐502‐3p expression is elevated in AD CSF exosomes, AD serum exosomes, AD B‐lymphocytes and Aβ‐ and Tau‐treated cells.
2. MiR‐501‐3p and miR‐502‐3p are correlated with amyloid plaque and NFT tangle density in specific brain regions.
3. MiR‐501‐3p and miR‐502‐3p are highly expressed in neurons and astrocytes, suggesting that these cells are the source of miRNA secretion.
4. MiR‐501‐3p and miR‐502‐3p could be a promising biomarker panel for AD.

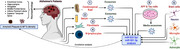

## 1 BACKGROUND

Alzheimer's disease (AD) is a progressive, neurodegenerative disorder that remains the most common cause of dementia, accounting for 60–80% of all cases.^1^ Approximately 6.7 million people in the United States over the age of 65 years are currently living with AD. By 2060, this is projected to grow to 13.8 million.^1^ In AD, amyloid beta (Aβ) plaques, composed of Aβ peptides, and neurofibrillary tangles (NFTs), composed of hyperphosphorylated tau (p‐tau), accumulate in the brain and trigger proinflammatory events that cause neurodegeneration.^2^, ^3^ The basal forebrain cholinergic neurons are among the affected cells, which causes an acetylcholine deficiency. This plays a major role in cognitive decline and is considered to be a hallmark of AD.^4^, ^5^ Currently, no precise biomarkers or curative treatment exists for AD. United States Food and Drug Administration‐approved medications remain limited, and most only provide temporary symptomatic benefits.^6^, ^7^, ^8^, ^9^

One of the diagnostic tools we have for AD is the cerebrospinal fluid (CSF) analysis of p‐tau, Aβ42 and total Tau protein content, which reflects amyloidosis and tauopathy in the brain.^10^ However, for a successful diagnosis, the disease would already have to be relatively advanced.^2^ Early diagnosis of AD leads to proactive treatment of the disease, thus delaying the onset of dementia. This will not only improve prognosis, but it will also result in substantial cost savings to healthcare systems.^11^ For these reasons, searching for novel biomarkers has been a large focus of AD research in recent history.^12^, ^13^ One of the most promising biomarkers that have emerged is microRNA (miRNA).^3^, ^14^, ^15^, ^16^, ^17^, ^18^ The role of miRNAs as biomarkers is currently being studied in many diseases, including cancers, autoimmune conditions and neurodegenerative disorders.^3^, ^17^, ^18^, ^19^, ^20^, ^21^, ^22^, ^23^, ^24^, ^25^ Previously, we reviewed the involvement of different miRNAs in ageing and senescence.^26^ Many miRNAs have been found to have differential expression in serum and CSF of AD patients, suggesting their possible potential as biomarkers.^27^ Among them, we had previously studied miR‐455‐3p extensively regarding its role in AD and its potential as a peripheral biomarker.^28^, ^29^, ^30^, ^31^, ^32^ Despite the numerous candidates, none of these potential miRNA biomarkers have yet passed the clinical trials stage. Therefore, it is important to continue developing our understanding of the importance of miRNAs in a clinical setting.

Recently, our laboratory discovered the high fold expression of miRNA‐501‐3p (miR‐501‐3p) and miRNA‐502‐3p (miR‐502‐3p) in the synaptosomes (synapses) of AD brains relative to unaffected controls (UC).^33^, ^34^ In this study, we found that miR‐501‐3p and miR‐502‐3p were among the top miRNAs sequentially overexpressed as AD progressed through the Braak stages. We also confirmed high expression levels of miR‐501‐3p and miR‐502‐3p in the synaptosomes of APP and Tau transgenic mice.^33^ Further characterisation of these two synapse‐specific miRNAs showed that they regulate the GABAergic synaptic pathway by reducing *GABRA1* expression. Disruption of this pathway plays a role in the synaptic dysregulation seen in AD, which was further supported by the increasing expression of miR‐501‐3p and miR‐502‐3p with worsening AD stages.^33^ Our recent findings investigated the roles of miR‐502‐3p in the modulation of GABAergic synapse function in AD.^35^, ^36^ Following, we further explored the possible roles of miR‐501‐3p and miR‐502‐3p in various human diseases, including AD.^3^ Since these miRNAs are differentially expressed in many disorders, they could potentially serve as biomarkers for AD. Therefore, it is important to determine the status of miR‐501‐3p and miR‐502‐3p in the periphery, such as CSF or serum, to elucidate if differential levels of these miRNAs could reflect AD progression. Here, we aim to explore the biomarker potential of miR‐501‐3p and miR‐502‐3p by assessing their presence in central and peripheral circulation. We analysed them in AD CSF, serum, fibroblasts and B‐lymphocytes. We also investigated the cellular and extracellular status of miR‐501‐3p and miR‐502‐3p in response to Aβ and tau pathology using cell culture studies. Our study on various AD sources unveiled strong biomarker capabilities of miR‐501‐3p and miR‐502‐3p for AD.

## 2 METHODS

### 2.1 CSF samples

CSF samples from AD patients and UCs were obtained from NIH NeuroBioBank Center‐Mount Sinai NIH Brain and Tissue Repository, 130 West Kingsbridge Road Bronx, NY59. Thirty‐six CSF samples, including AD patients (*n* = 26), age and sex‐matched UC samples (*n* = 10), were received from NIH NeuroBioBank. Demographic details such as: age, tissue type (CSF/serum), manner of death, sex, race, age, PMI (post‐mortem interval) and diagnosis of the AD and UC CSF samples are summarised in Table S1. The samples were collected during the period of 1988 to 2022. We received the neuropathology reports from the NIH NeuroBioBank corresponding to each CSF sample used in our study. The neuropathological details of the samples such as plaques and tangle density in the different brain regions are summarised in Table S2. The amyloid plaques and NFT density were determined in the entorhinal cortex, amygdala, hippocampus, middle frontal gyrus, superior temporal gyrus and inferior parietal lobule areas following neuropathology reports. The plaques and tangles distribution are categorised as none, sparse, moderate, frequent/severe based on the plaques and tangle density. The study was conducted at the Center of Emphasis in Neuroscience, Department of Molecular and Translational Medicine, Texas Tech University Health Sciences Center, El Paso, and Institutional Biosafety Committee (IBC protocol #22008) approved the study protocol for the use of human biological samples obtained from NIH NeuroBioBanks. The NIH NeuroBioBanks mentioned above are operated under their respective institution's IRB approvals and obtained written informed consent from the donors or their family members.

### 2.2 Serum samples

Serum samples from AD patients and UC were obtained from Texas Alzheimer's Research and Care Consortium (TARCC). Fifty‐nine serum samples, including AD patients (*n* = 25), mild cognitive impairment (*n* = 15), age and sex‐matched UC samples (*n* = 19), were received from TARCC. Table S3 summarises the samples’ age, sex, race, Mini‐Mental State Examination score, APOE information (when available) and total tau (when available).

### 2.3 Fibroblasts and B‐lymphocyte samples

Fibroblasts and B‐lymphocyte samples were obtained from the Coriell Institute of Medical Research, 403 Haddon Ave, Camden, NJ. Three levels of disease pathology differentiation existed within the fibroblast and B‐lymphocyte samples: familial AD (fAD), sporadic AD (sAD) and UC samples. Eighteen fibroblast samples, including fAD (*n* = 4), sAD (*n* = 6) and UCs (*n* = 8) were received from the Coriell Institute of Medical Research. Additionally, we received twenty‐two B‐lymphocyte samples, including fAD (*n* = 6), sAD (*n* = 6) and UCs (*n* = 10) were received from the from the same institution. Demographic information for fibroblast and B‐lymphocyte samples, such as sex, age, biopsy source, tissue type, race and disease status are summarised in Tables S4 and S5, respectively.

### 2.4 SH‐SY5Y cells and plasmid transfection

Human neuroblastoma (SH‐SY5Y) cells are routinely maintained in our laboratory.^30^ Cells were cultured in Dulbecco's modified eagle medium (DMEM; Gibco™) supplemented with Ham's F‐12 nutrient media, exosomes‐depleted 10% foetal bovine serum (FBS) and 1× antibiotic–antimycotic (Gibco) at 37°C in a humidified atmosphere containing 5% CO_2_. Cells were transfected with control plasmid (VB010000‐9829sne; Vector builder), APP plasmid (pCAX APP Swe/Ind; AddgGene) and Tau plasmid (pRK5–EGFp–Tau E14 P301L; AddGene) using Lipofectamine 3000 following the manufacturer protocol (ThermoFisher Scientific, USA). The complete details of plasmids are provided in Figure S1. At 48 h post‐transfection, the spent media (3 mL) were collected from control, APP and Tau plasmids transfected cells for exosome isolation.

### 2.5 Exosome isolation

Exosomes were extracted from CSF, serum and cell culture media using specific kits following the manufacturer's instructions. Exosomes were isolated from CSF samples using the total exosome isolation reagent (from other body fluids) kit (Catalog Number: 4484453; Invitrogen, USA). Serum exosomes were isolated from the serum samples using total exosome isolation reagent (from serum) kit (Catalog Number: 4478360; Invitrogen). Exosomes from the cell culture media were isolated by using the total exosome isolation reagent (from cell culture media) kit (Catalog Number: 4478359; Invitrogen).

### 2.6 Transmission electron microscopy of exosomes

Exosomes were isolated from CSF, serum, and cell culture media, and their morphology was characterised by transmission electron microscopy (TEM) analysis.^32^ Exosome pellets were washed with 1XPBS and dissolved in a fixative solution (8% Glutaraldehyde, 16% paraformaldehyde and 0.2 M sodium cacodylate buffer) for 1 h at room temperature. Samples were centrifuged at 300×*g* for 10 min, and the resulting exosome pellet underwent electron microscopy at the imaging core facility at Texas Tech University, Lubbock, Texas, USA.^31^

### 2.7 Immunoblotting analysis for exosome marker proteins

For immunoblotting analysis, exosome pellets isolated from CSF, serum and cell media were suspended in RIPA buffer (Thermo Scientific, USA) with (1×) protease inhibitor and EDTA. The samples were sonicated (Amplitude 50%, Pulse 2 sec on/off) for 10 s on ice. Exosome proteins were quantified by bicinchoninic acid assay method. The 40 µg of exosome protein were mixed in a 4:1 (v/v) ratio with 4× LDS sample loading buffer (Novex) and subjected to SDS‐PAGE analysis. The electrophoresis was carried out in mini protein tetra cell (BioRad, USA) at 100 V for 1 h. Transfer sandwiches were prepared with an anode‐facing gel and a cathode‐facing PVDF membrane for each blot. Protein transfer was carried out for 10 min in transfer buffer using the trans‐blot turbo (BioRad) transfer system. Thereafter, blots (PVDF‐membranes) were blocked with 5% (w/v) bovine serum albumin (BSA) in tris‐buffer saline with 0.1% tween (1× TBS‐T). Blot(s) were incubated overnight with primary antibodies specific to biomarkers, that is, CD9 (Mouse; 1:500), CD63 (Mouse; 1:500), TSG101 (Mouse; 1:500), NeuN (Mouse; 1:500), GFAP (Mouse; 1:1000), IBA1 (Mouse; 1:1000), APP (Rabbit; 1:1000), Tau (Mouse; 1:1000) and GAPDH (Rabbit; 1:3000) prepared in BSA (5% w/v; 10 mL) at 4°C. The complete details of antibodies and their dilutions are provided in Table S6. Thereafter, blot(s) were washed three times with TBS‐T buffer and incubated for 2 h at room temperature with secondary antibodies, that is, anti‐mouse/anti‐rabbit IgG‐peroxidase (Sigma; 1:10 000 prepared in BSA 5% w/v). After washing the blot(s) five times with TBS‐T buffer, the protein bands labelled with secondary antibodies were detected using the Clarity™ enhanced chemiluminescence (ECL; BioRad) western blotting substrate. Blot(s) were then visualised by exposing it to dark conditions in a luminescent image analyser (Amersham imager 680; GE Healthcare Bio‐Sciences, Sweden).^32^

### 2.8 Particle size analysis of exosomes

The particle sizes of micro‐vesicles isolated from CSF, serum and cell culture media were determined by dynamic light scattering (DLS) analysis using the NanoSight (Malvern zetasizer, Worcerestershire, UK). The exosome pellets were diluted with 1× phosphate buffer saline (1:100) and processed for exosome particle size distribution analysis. The particle size and density were recorded along all the sample groups.^37^

### 2.9 Exosome RNA isolation

The exosome pellets from CSF, serum, cell media were suspended in Trizol (500 µL) for total RNA isolation. Following the addition of chloroform (100 µL), samples were vortexed and incubated at room temperature for 10 min and centrifuged at 12 000×*g* for 30 min at 4°C. Aqueous phase was transferred into fresh eppendorf tubes. Thereafter, isopropanol (250 µL) was added to each sample(s) and incubated overnight at −20°C. RNA pellets were collected by centrifugation at 12 000×*g* for 30 min at 4°C and washed with ethanol (500 µL; 75% v/v). Samples were again centrifuged at 12 000×*g* for 30 min at 4°C. After washing ethanol was removed, samples were air dried and suspended in DEPC‐treated water (12 µL). The RNA concentration (ng/µL) of each sample was determined by nano‐drop 2000 spectrophotometer (Thermo Scientific). cDNA was synthesised from total RNA (2 µg) including miRNA, in two steps process: polyadenylation followed by reverse transcription using First strand cDNA synthesis kit (Agilent Technologies, USA; Catalog Number: 600036) in a thermal cycler (Applied Biosystem 9902; USA) according to the manufacturer's protocol.^32^

### 2.10 Quantitative real‐time PCR analysis of miR‐501‐3p and miR‐502‐3p

The expressions of miR‐501‐3p and miR‐502‐3p were quantified by qRT‐PCR. qRT‐PCR was performed by preparing a reaction mixture containing 1 µL of miR‐501‐3p or miR‐502‐3p specific forward primer (10 µm), 1 µL of a universal reverse primer (3.125 µM; Agilent Technologies Inc.), 5 µL of 2X SYBR green PCR master mix (Kappa Biosystems) and 1 µL of cDNA. RNase‐free water was added to the mixture to bring the total volume to 10 µL. Primers used in the current study were commercially synthesised from Integrated DNA Technologies, Iowa, USA (Table S7). Reactions were run in duplicate using the LightCycler® 96 (Roche, USA). U6 snRNA served as the internal control. MiRNA fold changes were calculated using (2^−ΔΔct^) method.^29^, ^30^, ^32^, ^38^ Where, ΔCt = (Ct value of miR‐501/502‐3p − Ct value of U6 snRNA) ΔΔCt = (ΔCt value of miR‐501/502‐3p in AD patients − ΔCt value of UC samples).

### 2.11 MiR‐501‐3p and miR‐502‐3p analysis with amyloid plaques and NFT density in affected brain regions

We evaluated the neuropathology reports of AD CSF samples in relation to amyloid plaques and NFT density across affected brain regions. The amyloid plaques and NFT densities were categorised as none, sparse, moderate and frequent/severe. Fold changes of miR‐501‐3p and miR‐502‐3p in CSF samples were analysed and correlated with neuropathology reports provided by the NIH NeuroBioBanks, specifically assessing amyloid plaques and NFT density across different brain regions, including the entorhinal cortex, hippocampus, amygdala, middle frontal gyrus, inferior parietal lobule and superior temporal gyrus.

### 2.12 MiR‐501‐3p and miR‐502‐3p expression analysis in brain cells

To assess the miR‐501‐3p and miR‐502‐3p expression in both human and mouse brain cell types, human cortical neurons (HCN‐2/Cat # CRL‐3592), human microglia (HMC3/Cat # CRL‐3304), human astrocytes (CCF‐STTG1/Cat # CRL‐1718), mouse microglia (SIM‐A9/Cat # CRL‐3265) and mouse astrocytes (C8‐D1A/Cat # CRL‐2541) were obtained from the American type culture collection (ATCC, Manassas, USA), and mouse hippocampal neurons (mHippoE‐14/Cat # CLU‐198), from the Cedarlane Laboratories (North Carolina, USA). The complete details and origin of cell lines are provided in the Table S8. All cell lines were cultured in their recommended growth media, supplemented with 10% FBS and 1% penicillin–streptomycin–neomycin. All cell lines were maintained at 37°C in a humidified incubator with 5% CO₂. Total RNA, including miRNAs, was extracted from cell pellets using Trizol method described above. cDNA synthesis and qRT‐PCR were performed following the protocol as described above. U6 snRNA was used for normalisation, and relative miRNA expression was calculated using the 2^−ΔCt method.^29^, ^30^, ^32^, ^38^ Where, ΔCt = (Ct value gene of miR‐501‐3p/miR‐502‐3p − Ct value of U6 snRNA).

### 2.13 Statistical analysis

Statistical parameters were calculated using GraphPad Prism software, v8 (La Jolla, CA, USA) and OriginPro 2024b (Northampton, MA, USA). Results are reported as mean ± SD and median ± SEM. The qRT‐PCR results were analysed by Students *t*‐tests (unpaired t test) comparing the miR‐501‐3p and miR‐502‐3p expression in AD versus UC CSF exosomes. The ordinary one‐way ANOVA was implied to compare the miR‐501‐3p and miR‐502‐3p expression in UC serum versus MCI serum versus AD serum exosomes. The unpaired t test was applied to compare the miR‐501‐3p and miR‐502‐3p expression in UC fibroblast versus fAD fibroblasts and sAD fibroblasts. Similarly, unpaired *t*‐test was applied to compare the miR‐501‐3p and miR‐502‐3p expression in UC B‐lymphocytes versus fAD B‐lymphocytes and sAD B‐lymphocytes. Further, unpaired *t* test was used to compare the miR‐501‐3p and miR‐502‐3p expression in control versus APP and control versus Tau cells. The ordinary one‐way ANOVA was implied to compare the miR‐501‐3p and miR‐502‐3p expression in neurons versus microglia versus astrocytes. *p* < .05 was considered statistically significant.

## 3 RESULTS

**FIGURE 1:**
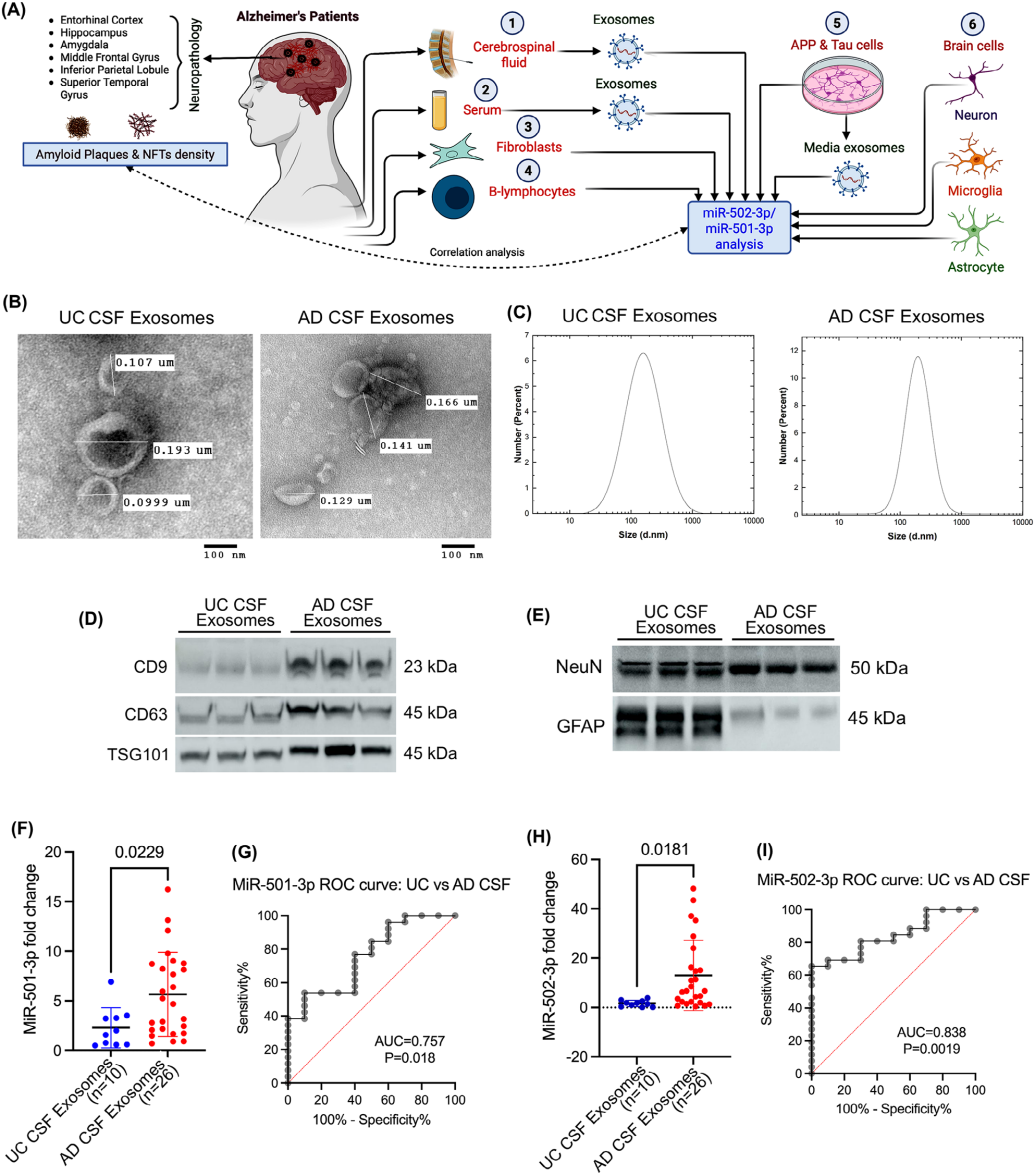
Status of miR‐501‐3p and miR‐502‐3p in AD and UC CSF samples. (A) Brief study plan: Exosomes were extracted from AD and UC CSF, serum, APP and Tau plasmid transfected cells. Afterward, miR‐501‐3p and miR‐502‐3p expression were quantified from these exosomes. Additionally, miR‐501‐3p and miR‐502‐3p quantification was also performed on AD and UC fibroblasts and B‐lymphocytes as well as on brain cells. Expression of these two miRNAs from CSF samples was then correlated with the neuropathology report of the same samples regarding the amyloid plaques and NFT density in different brain regions. (B) TEM images of exosomes from UC and AD CSF samples (scale bar: 100 nm). (C) Particle size analysis of exosomes using DLS in UC and AD CSF samples. (D) Immunoblotting of exosome marker proteins CD9, CD63 and TSG101 in UC and AD CSF samples (*n* = 3). (E) Immunoblotting of neuronal marker, NeuN and astrocytic marker, GFAP in UC and AD CSF samples (*n* = 3). (F) Expression of miR‐501‐3p in UC CSF exosomes (*n* = 10) compared with AD CSF exosomes (*n* = 26) (*t*‐test, *p* = .0229). (G) ROC curve analysis of miR‐501‐3p expression in UC versus AD CSF exosomes (AUC = 0.757, *p* = .018). (H) Expression of miR‐502‐3p in UC CSF exosomes (*n* = 10) compared with AD CSF exosomes (*n* = 26) (*t*‐test, *p* = .0181). (I) ROC curve analysis of miR‐502‐3p expression in UC versus AD CSF exosomes (AUC = 0.838, *p* = .0019). Data are presented as mean ± SEM.

We have divided the present investigation into six different studies. Figure 1A briefly outlined the overall work plan of the study. We used different AD sources to investigate the biomarker potential of miR‐501‐3p and miR‐502‐3p in relation to Aβ and Tau pathology: (1) CSF, (2) serum, (3) fibroblasts, (4) B‐lymphocytes, (5) APP and Tau plasmid transfected cells and (6) brain cells.

### 3.1 CSF study

#### 3.1.1 Characterisation of CSF exosomes in UC and AD samples

We begin by evaluating CSF samples obtained from UC and AD patients. First, the exosomes were isolated from the CSF samples. The isolated exosomes were then characterised by TEM, immunoblotting and DLS analysis. The TEM analysis showed the cup‐shaped ultrastructure of exosomes in both UC and AD CSF samples (Figure 1B). The exosome vesicle size ranged from 0.101 to 0.185 µm in both UC and AD. We did not see any significant size differences between the exosomes in UC versus AD. Next, we determined the micro‐vesicle size range using the DLS analysis. The UC CSF exosomes ranged from 10 to 800 nm with peaks of around 300 nm, and AD CSF exosomes ranged from 90 to 700 nm with peaks of around 300 nm (Figure 1C). DLS analysis also revealed a similar size distribution based on the highest intensity peak for number of exosomes. Next, we characterised the exosomes by assessing the expression of exosome marker proteins CD9, CD63 and TSG101. The immunoblotting analysis showed detectable levels of CD9, CD63 and TSG101 proteins (Figure 1D). Furthermore, we aimed to determine which brain cells produced the isolated exosomes. Immunoblotting analysis of brain cell markers showed a detectable level of neuronal marker NeuN and astrocyte marker GFAP (Figure 1E). We performed the immunoblotting analysis of microglial marker IBA1 but did not see any detectable expression of IBA in CSF exosomes. These results confirmed successful exosome extraction from the CSF and suggested that the exosomes released into the CSF contributed by both neurons and astrocytes.

#### 3.1.2 MiR‐501‐3p and miR‐502‐3p expression analysis in CSF exosomes

To determine the biomarker potential of miR‐501‐3p and miR‐502‐3p in CSF, we assessed their expression levels in the exosomes isolated from UC and AD CSF samples. qRT‐PCR analysis of miR‐501‐3p showed a significantly (*p* = .0229) higher expression of miR‐501‐3p in AD CSF exosomes relative to UC CSF exosomes (Figure 1F). Afterward, to assess the diagnostic value of miR‐501‐3p fold‐change in UC versus AD samples, we conducted a receiver operating characteristic (ROC) curve analysis. Our results showed a significant area under the curve (AUC) value for miR‐501‐3p fold change (AUC = 0.757, *p* = .018) (Figure 1G).

Similarly, we analysed the miR‐502‐3p expression in UC and AD CSF exosome samples. qRT‐PCR analysis of miR‐502‐3p also showed a significantly (*p* = .0181) higher expression of miR‐502‐3p in AD CSF exosomes relative to UC CSF exosomes (Figure 1H). We conducted a ROC curve analysis for miR‐502‐3p fold change, which indicated a significant AUC value for miR‐502‐3p fold change (AUC = 0.838; *p* = .0019) in UC CSF exosomes versus AD CSF exosomes (Figure 1I). Finally, we conducted a ROC curve analysis for combined miR‐501‐3p and miR‐502‐3p fold change, which revealed a significant AUC value (AUC = 0.797; *p* < .0001) in UC CSF exosomes versus AD CSF exosomes (Figure S1A). These observations suggest that both miRNAs could be potential synapse‐associated exosome biomarkers for AD and show their discriminating power between UC and AD.

#### 3.1.3 MiR‐501‐3p expression relative to amyloid plaque and NFT density in affected brain regions

**FIGURE 2:**
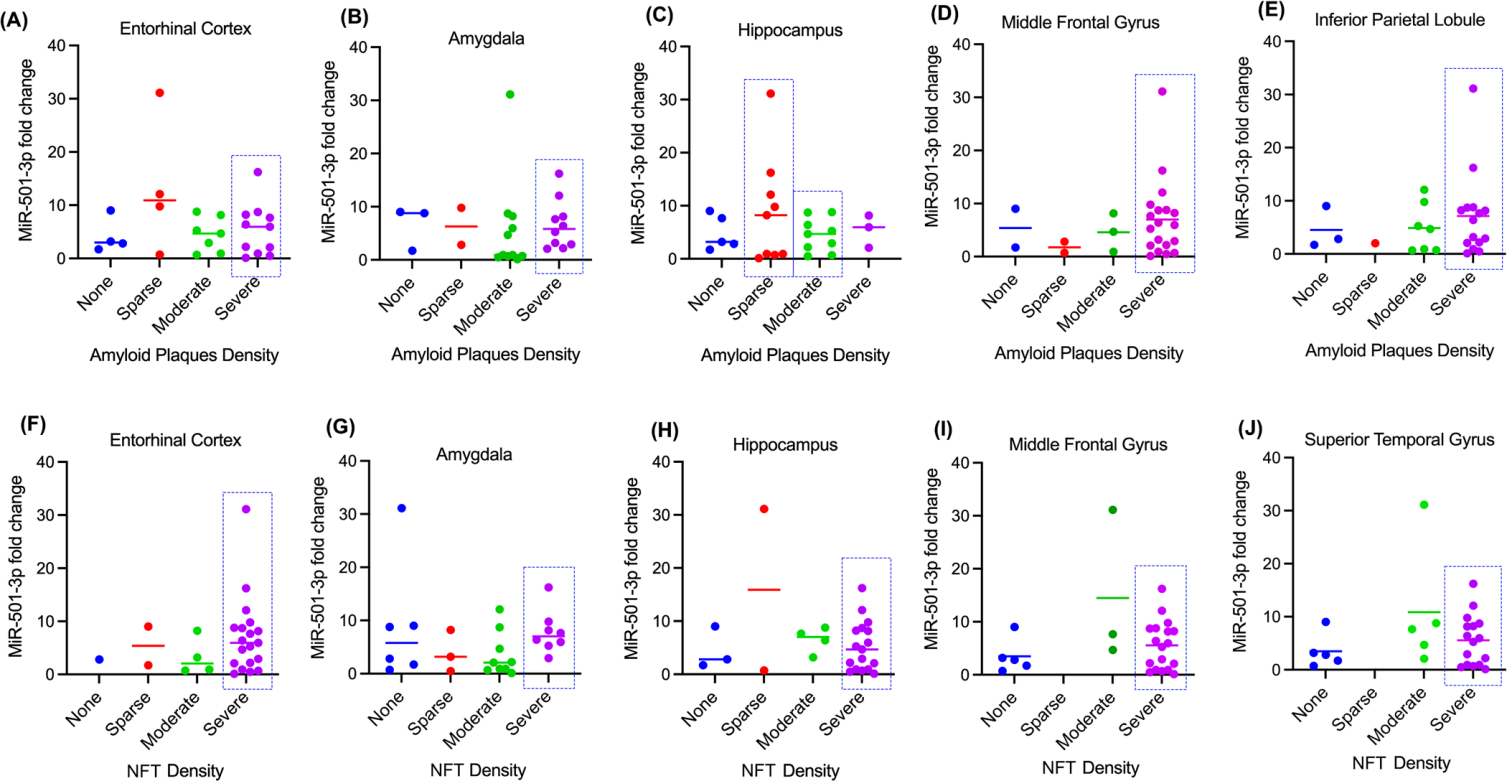
Relationship of miR‐501‐3p expression and the neuropathology in AD CSF samples. MiR‐501‐3p expression was correlated with amyloid plaque density in the (A) entorhinal cortex, (B) amygdala, (C) hippocampus, (D) middle frontal gyrus and (E) inferior parietal lobule. MiR‐501‐3p expression was also correlated with NFT density in the (F) entorhinal cortex, (G) amygdala, (H) hippocampus, (I) middle frontal gyrus and (J) superior temporal gyrus. The dotted box shows the categories with the highest number of samples and high average miR‐501‐3p fold change.

We wanted to investigate if there was a relationship between CSF miRNA fold change and AD pathology. To do so, we first compared miR‐501‐3p fold change with neuropathology report of amyloid plaque density in the entorhinal cortex, amygdala, hippocampus, middle frontal gyrus and inferior parietal lobule of the same patients (Figure 2A–E). Since there was a vastly variable distribution of patients in the different severity categories, we could only make qualitative observations. A majority (69%) of the patients had moderate (*n* = 7) or severe (*n* = 11) amyloid plaque density in the entorhinal cortex (Figure 2A). Aside from the sparse severity, the data show a positive correlation between average miR‐501‐3p fold change and amyloid plaque density in the entorhinal cortex (Figure 2A). Similarly, 81% of patients had moderate (*n* = 11) or severe (*n* = 10) amyloid plaque density in the amygdala (Figure 2B). No correlation can be observed between miR‐501‐3p fold change and plaque severity in the amygdala. Interestingly, only 46% of patients showed moderate (*n* = 9) or severe (*n* = 3) amyloid plaque density in the hippocampus (Figure 2C). Here, miR‐501‐3p fold change is most highly correlated with sparse amyloid plaque density (Figure 2C). Next, 73% of patients had severe amyloid plaque density (*n* = 19), and 8% of patients had no amyloid plaque density (*n* = 2) in the middle frontal gyrus (Figure 2D). Here, miR‐501‐3p fold change is most highly correlated with severe amyloid plaque density (Figure 2D). Finally, 85% of patients had moderate (*n* = 7) or severe (*n* = 15) amyloid plaque density in the inferior parietal lobule, while 12% had no plaques (*n* = 3) (Figure 2E). For this brain region, miR‐501‐3p fold change is most highly correlated with severe amyloid plaque density (Figure 2E).

Next, we evaluated miR‐501‐3p level with NFT density in the entorhinal cortex, amygdala, hippocampus, middle frontal gyrus and superior temporal gyrus in the AD brain (Figure 2F–J). 73% of patients had severe (*n* = 19) NFT density in the entorhinal cortex (Figure 2F). While the data show that miR‐501‐3p fold change is most highly correlated with severe NFT density, it is difficult to confirm this as there are very few patients in the other severity categories (Figure 2F). Next, a majority 65% of the patients had moderate (*n* = 9) or severe (*n* = 8) NFT density in the amygdala (Figure 2G). Here, miR‐501‐3p fold change is most highly correlated with severe NFT density (Figure 2G). Unlike with amyloid plaque density, a majority (65%) of the patients had severe NFT density in the hippocampus (Figure 2H). It is difficult to conclude which severity has the highest correlation with miR‐501‐3p fold change here because there are few patients in the other categories. Similar to amyloid plaque density, 69% of patients had severe (*n* = 18) NFT density in the middle frontal gyrus, and 19% of patients had no NFT density (*n* = 5) (Figure 2I). This bimodal distribution of patients makes it difficult to conclude correlation patterns with miR‐501‐3p fold change and NFT density in this region of the brain. Finally, there was also a bimodal distribution of NFT density in the superior temporal gyrus, with 62% of patients having severe (*n* = 16) NFT density and 19% of patients having no (*n* = 5) NFT density (Figure 2J). As with the middle frontal gyrus, it is difficult to conclude correlation patterns with this distribution. Overall, these results suggest that miR‐501‐3p expression in the CSF could reflect some degree of amyloid plaque density or NFT density in the brain; however, a study with a higher number of samples that is more evenly distributed among the different severities is necessary to explore this further.

#### 3.1.4 MiR‐502‐3p expression relative to amyloid plaque and NFT density in affected brain regions

**FIGURE 3:**
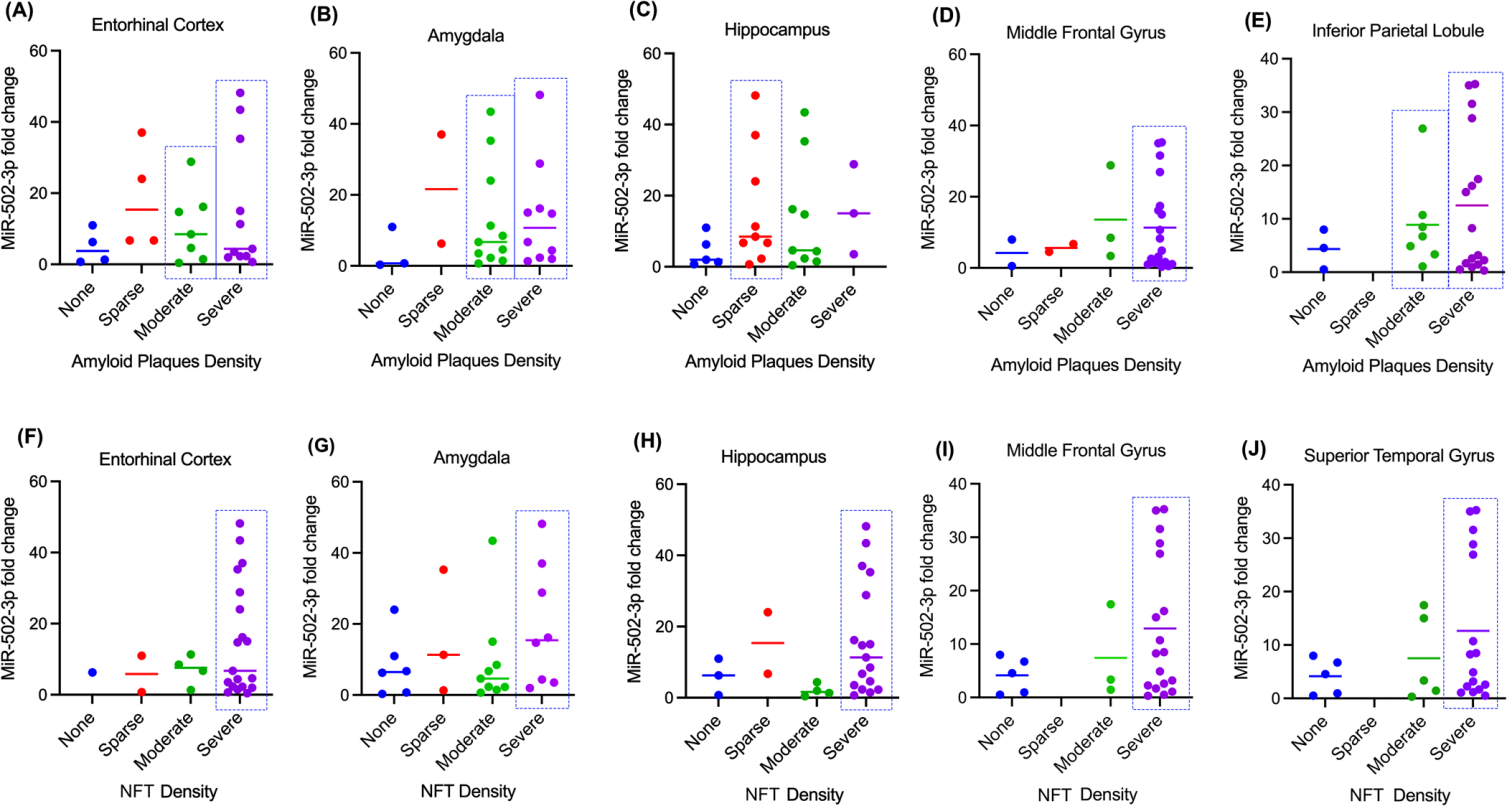
Relationship of miR‐502‐3p expression and the neuropathology in AD CSF samples. MiR‐502‐3p expression was correlated with amyloid plaque density in the (A) entorhinal cortex, (B) amygdala, (C) hippocampus, (D) middle frontal gyrus and (E) inferior parietal lobule. MiR‐502‐3p expression was also correlated with NFT density in the (F) entorhinal cortex, (G) amygdala, (H) hippocampus, (I) middle frontal gyrus and (J) superior temporal gyrus. The dotted box shows the categories with highest number of samples and high average miR‐502‐3p fold change.

Likewise, we also compared miR‐502‐3p fold change with amyloid plaque density in the entorhinal cortex, hippocampus, amygdala, middle frontal gyrus and inferior parietal lobule of the same patients (Figure 3A–E). The distribution of amyloid plaque severity among the patients is the same as discussed in the previous section (Figures 2A–E). In the entorhinal cortex, miR‐502‐3p fold change average is better correlated with moderate than severe amyloid plaque density, but the patients that have the highest miR‐502‐3p fold change have severe amyloid plaque density (Figure 3A). In the amygdala, miR‐502‐3p fold change average is better correlated with severe amyloid plaque density than moderate (Figure 3B). Interestingly, miR‐502‐3p fold change is better correlated with sparse amyloid plaque density than moderate (Figure 3C). While the fold change for severe amyloid plaque density is higher on average than sparse, there are only three patients in that group. In the middle frontal gyrus, miR‐502‐3p fold change average is more correlated with severe amyloid plaque density than the other groups (Figure 3D). Finally, miR‐502‐3p fold change is more correlated with severe amyloid plaque density than the other groups in the inferior parietal lobule (Figure 3E).

Next, we evaluated miR‐502‐3p level with NFT density in the entorhinal cortex, hippocampus, amygdala, middle frontal gyrus and superior temporal gyrus in the AD brain (Figure 3F–J). Like for amyloid plaque density, the distribution of NFT density severities among the patients is the same as discussed in the previous section (Figures 2F–J). In the entorhinal cortex, the average miR‐502‐3p fold change is similar between the different NFT density severities; however, none, sparse and moderate NFT density groups each have limited patients. As such, it is difficult to conclude the correlation here (Figure 3F). In the amygdala, miR‐502‐3p fold change is more correlated with severe NFT density than the other groups (Figure 3G). In the hippocampus, miR‐502‐3p average fold change is better correlated with severe NFT density than moderate or no NFT density (Figure 3H). While miR‐502‐3p average fold change is higher in sparse NFT density than severe, there are only two patients in that group. Similarly, miR‐502‐3p average is most correlated with severe NFT density in both the middle frontal gyrus and superior temporal gyrus (Figure 3I and J).

Except for the entorhinal cortex, each brain region analysed had severe amyloid plaque and NFT densities that most correlated with miR‐502‐3p average fold change. These results suggest that miR‐502‐3p expression in the CSF could reflect severe degrees of amyloid plaque density or NFT density in these brain regions. However, as stated in the section above, a study with a larger sample size must be conducted.

### 3.2 | Serum study

#### 3.2.1 Characterisation of serum exosomes in UC, MCI and AD samples

**FIGURE 4:**
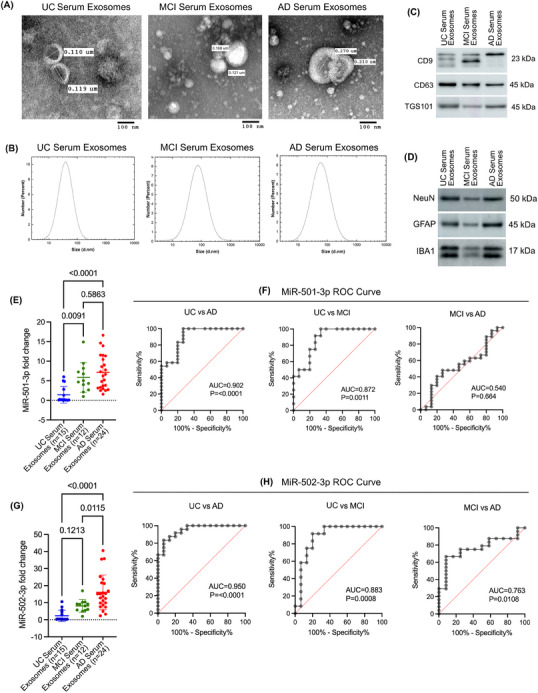
Characterisation of miR‐501‐3p and miR‐502‐3p in UC, MCI and AD serum exosome samples. (A) TEM images of exosomes from UC, MCI and AD serum samples (scale bar: 100 nm). (B) Particle size analysis of exosomes using DLS in UC, MCI and AD serum samples. (C) Immunoblotting of exosome marker proteins CD9, CD63 and TSG101 in UC, MCI and AD serum exosomes. (D) Immunoblotting of neuronal marker, NeuN, astrocytic marker, GFAP and microglial marker, IBA1 in UC, MCI and AD serum exosomes. (E) Expression of miR‐501‐3p in UC serum exosomes (*n* = 15) compared with MCI serum exosomes (*n* = 12, *t*‐test, *p* = .0091) and AD serum exosomes (*n* = 24, *t*‐test, *p* < .0001). Expression between MCI and AD serum exosomes was also compared (*t*‐test, *p* = .5863). (F) ROC curve analysis of miR‐501‐3p expression in UC versus AD serum exosomes (AUC = 0.902, *p* < .0001), UC versus MCI serum exosomes (AUC = 0.872, *p* = .0011) and MCI versus AD serum exosomes (AUC = 0.540, *p* = .664). (G) Expression of miR‐502‐3p in UC serum exosomes (*n* = 15) compared with MCI serum exosomes (*n* = 12, *t*‐test, *p* = .1213) and AD serum exosomes (*n* = 24, *t*‐test, *p* < .0001). Expression between MCI and AD serum exosomes was also compared (*t*‐test, *p* = .0115). (H) ROC curve analysis of miR‐502‐3p expression in UC versus AD serum exosomes (AUC = 0.950, *p* < .0001), UC versus MCI serum exosomes (AUC = 0.883, *p* = .0008) and MCI versus AD serum exosomes (AUC = 0.763, *p* = .0108). Data are presented as mean ± SEM.

As with the CSF samples, the exosomes were isolated from UC, MCI and AD serum samples. The isolated exosomes were characterised by TEM, immunoblotting and DLS analysis. The TEM analysis revealed the cup‐shaped ultrastructure of exosomes in UC, MCI and AD samples (Figure 4A). Furthermore, the exosome vesicle size ranged from 0.110 to 0.119 µm in UC, 0.121 to 0.168 µm in MCI, and 0.210 to 0.270 µm in AD. Next, we performed a DLS analysis to characterise the particle size further. The UC serum exosomes ranged from 10 to 110 nm with peaks of around 40 nm; MCI serum exosomes ranged from 10 to 200 nm with peaks of around 80 nm; and AD serum exosomes ranged from 10 to 300 nm with peaks of around 80 nm (Figure 4B). Interestingly, on average, MCI serum exosomes and AD serum exosomes were larger than UC exosomes. Next, we analysed the expression of exosome marker proteins CD9, CD63 and TGS101 in serum exosomes. The immunoblotting analysis showed detectable CD9, CD63 and TGS101 protein levels in the UC, MCI and AD samples (Figure 4C). Finally, to determine the origin of exosomes, we performed an immunoblotting analysis of several brain cell markers. This showed detectable levels of NeuN, GFAP and IBA1, suggesting that these exosomes could have been released into the serum from neurons, astrocytes and microglia (Figure 4D). Overall, these results confirm the successful extraction of exosomes from the serum samples.

#### 3.2.2 MiR‐501‐3p and miR‐502‐3p expression analysis in AD and MCI serum exosomes

To assess the potential of miR‐501‐3p and miR‐502‐3p as serum biomarkers, we evaluated their expression levels in the exosomes isolated from UC, MCI and AD serum samples. qRT‐PCR analysis showed higher levels of miR‐501‐3p and miR‐502‐3p in MCI serum exosomes relative to UC serum exosomes. Furthermore, we observed even higher levels of miR‐501‐3p and miR‐502‐3p in AD serum exosomes relative to MCI and UC serum exosomes. MiR‐501‐3p levels were significantly up‐regulated in MCI relative to UC (*p* = .0091) and in AD relative to UC (*p* ≤ .0001); however, miR‐501‐3p levels were not significantly higher in AD compared with MCI (*p* = .5863) (Figure 4E). Furthermore, we conducted a ROC curve analysis to assess the diagnostic value of miR‐501‐3p fold change in serum samples. Our results show a significant AUC for miR‐501‐3p fold change in UC versus AD (AUC = 0.902; *p* ≤ .0001), UC versus MCI (AUC = 0.872; *p* = .0011) and but not for MCI versus AD (AUC = 0.540; *p* = .664) (Figure 4F).

Interestingly, miR‐502‐3p levels were not significantly up‐regulated in MCI relative to UC (*p* = .1213); however, they were significantly up‐regulated in AD relative to UC (*p* ≤ .0001) and in AD compared with MCI (*p* = .0115) (Figure 4G). ROC analysis for miR‐502‐3p fold change showed a significant AUC for miR‐502‐3p fold change in UC versus AD (AUC = 0.950; *p* ≤ .0001); UC versus MCI (AUC = 0.883; *p* = .0008), and for MCI versus AD (AUC = 0.763; *p* = .0108) (Figure 4H). Finally, ROC analysis for combined miR‐501‐3p and miR‐502‐3p fold change in serum exosomes revealed a significant AUC for UC versus AD (AUC = 0.916; *p* < .0001), UC versus MCI (AUC = 0.880; *p* < .0001) and but not for MCI versus AD (AUC = 0.634; *p* = .052) (Figure S1B–D). These observations suggest that miR‐502‐3p and miR‐501‐3p could be potential serum biomarkers for AD, and ROC curve analysis showed their discriminating power between UC, MCI and AD.

### 3.3 | Fibroblast study

#### 3.3.1 MiR‐501‐3p and miR‐502‐3p expression analysis in fibroblast samples

**FIGURE 5:**
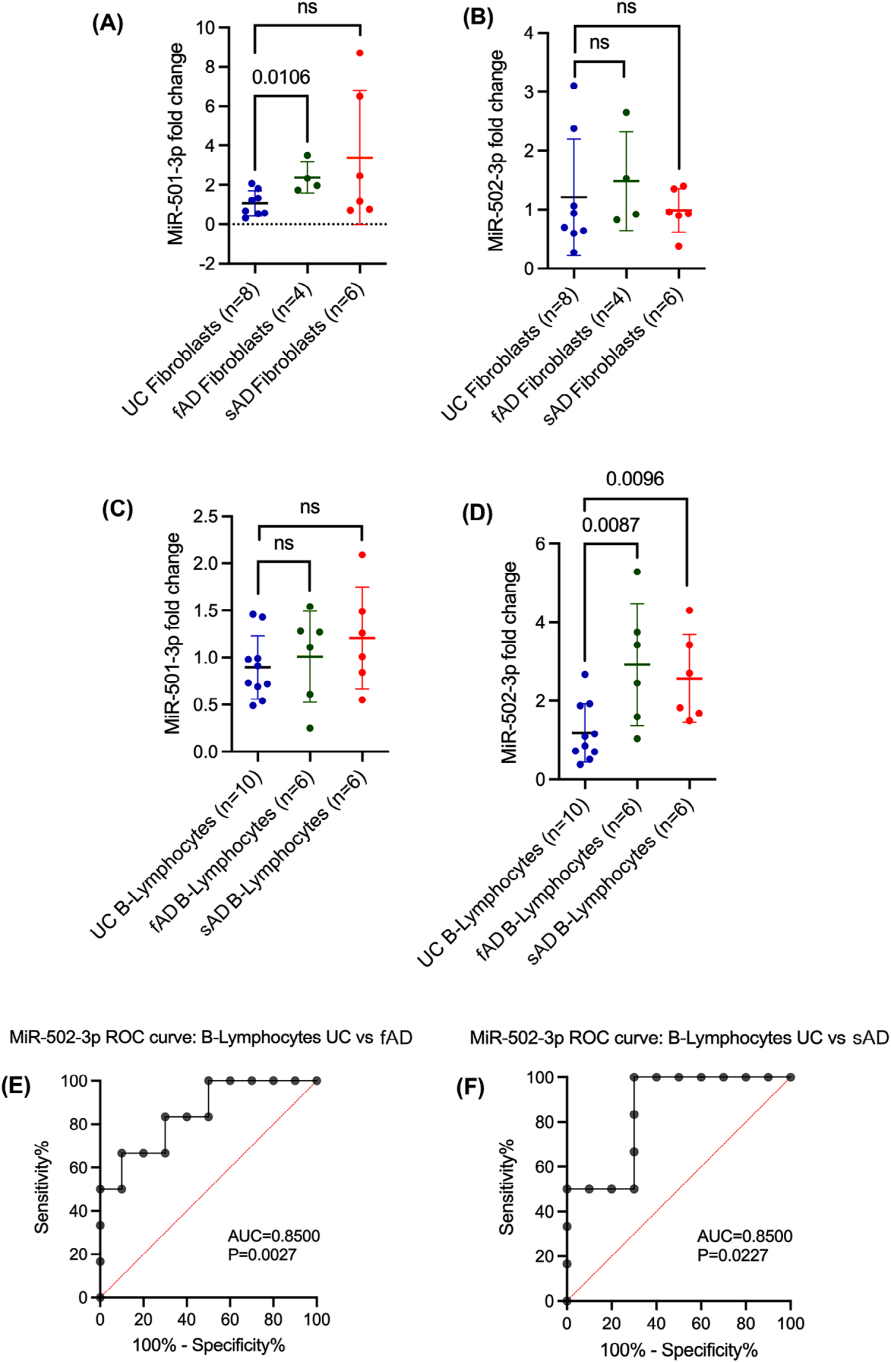
Expression of miR‐501‐3p and miR‐502‐3p in UC, fAD, sAD fibroblasts and B‐lymphocytes. (A) Expression of miR‐501‐3p in UC fibroblasts (*n* = 8) compared with fAD fibroblasts (*n* = 4, *t*‐test, *p* = .0106) and sAD fibroblasts (*n* = 6, *t*‐test, ns). (B) Expression of miR‐502‐3p in UC fibroblasts (*n* = 8) compared with fAD fibroblasts (*n* = 4, *t*‐test, *p* = ns) and sAD fibroblasts (*n* = 6, *t*‐test, ns). (C) Expression of miR‐501‐3p in UC B‐lymphocytes (*n* = 10) compared with fAD B‐lymphocytes (*n* = 6, *t*‐test, ns) and sAD B‐lymphocytes (*n* = 6, *t*‐test, ns). (D) Expression of miR‐502‐3p in UC B‐lymphocytes (*n* = 10) compared with fAD B‐lymphocytes (*n* = 6, *t*‐test, *p* = .0087) and sAD B‐lymphocytes (*n* = 6, *t*‐test, *p* = .0096). (E) ROC curve analysis of miR‐502‐3p expression in UC versus fAD B‐lymphocytes (AUC = 0.8500, *p* = .0027). (F) ROC curve analysis of miR‐502‐3p expression in UC versus sAD B‐lymphocytes (AUC = 0.8500, *p* = .0227). Data are presented as mean ± SEM. ns: not significant.

In addition to peripheral biomarkers, we were interested in investigating how miR‐501‐3p and miR‐502‐3p changed in other cells of the body. As such, we evaluated miR‐501‐3p and miR‐502‐3p levels in fibroblasts generated from patients with fAD (*n* = 4), sAD (*n* = 6) and UC (*n* = 8). MiR‐501‐3p levels were significantly up‐regulated in fAD relative to UC (*p* = .0106); however, they were not significantly increased in sAD relative to UC (Figure 5A). MiR‐502‐3p levels were not significantly different between UC, fAD and sAD (Figure 5B).

### 3.4 | B‐lymphocyte study

#### 3.4.1 MiR‐501‐3p and miR‐502‐3p expression in analysis of B‐lymphocyte samples

Further, we assessed miR‐501‐3p and miR‐502‐3p levels in B‐lymphocytes obtained from patients with fAD (*n* = 6), sAD (*n* = 6) and UC (n = 10). MiR‐501‐3p levels were not significantly different between UC, fAD and sAD (Figure 5C). MiR‐502‐3p levels were significantly up‐regulated in fAD relative to UC (*p* = .0087) and in sAD relative to UC (*p* = .0096) (Figure 5D). Additionally, we conducted a ROC curve analysis to assess the diagnostic value of miR‐502‐3p fold change in B‐lymphocytes. Our results show a significant AUC for miR‐502‐3p fold change in UC versus fAD (AUC = 0.8500; *p* = .0027) (Figure 5E) and UC versus sAD (AUC = 0.8500; *p* = .0227) (Figure 5F). The B‐lymphocyte data further corroborate the circulatory biomarker nature of miR‐502‐3p in AD.

### 3.5 | Cells Study

#### 3.5.1 APP and tau plasmids expression in human neuroblastoma cells

**FIGURE 6:**
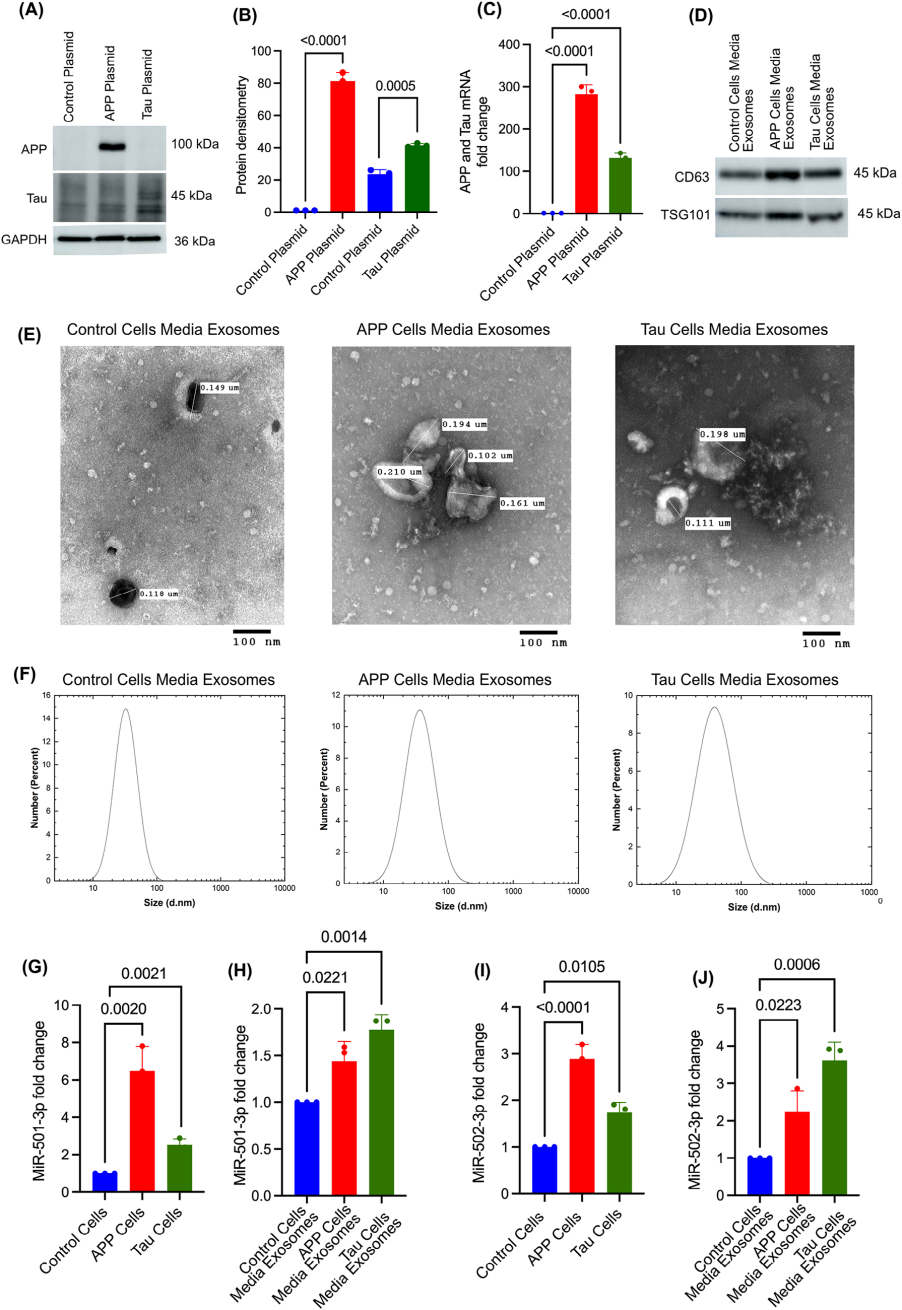
Status of miR‐501‐3p and miR‐502‐3p in APP plasmid‐transfected and Tau plasmid‐transfected cells and media exosomes. (A) Immunoblotting analysis of APP, Tau and GAPDH proteins in SH‐SY5Y cells transfected with control, APP and Tau plasmids. (B) APP densitometry analysis in control plasmid‐transfected cells versus APP‐transfected cells (*t*‐test, *p* < .0001) and Tau densitometry in control plasmid‐transfected cells versus Tau‐transfected cells (*t*‐test, *p* = .0005). (C) APP mRNA fold change in control plasmid‐transfected cells versus APP‐transfected cells (*t*‐test, *p* < .0001) and Tau mRNA fold change in control plasmid‐transfected cells versus Tau‐transfected cells (*t*‐test, *p* < .0001). (D) Immunoblotting analysis of exosome marker proteins CD63 and TSG101 in control, APP and Tau plasmids‐transfected cells media exosomes. (E) TEM images of exosomes from control plasmid‐transfected cell media, APP plasmid‐transfected cell media and Tau plasmid‐transfected cell media (scale bar: 100 nm). (F) Particle size analysis of exosomes in control, APP and Tau‐plasmids transfected SH‐SY5Y cells media exosomes using DLS. (G) Expression of miR‐501‐3p in control plasmid‐transfected cells compared with APP plasmid‐transfected cells (*t*‐test, *p* = .0020) and Tau plasmid‐transfected cells (*t*‐test, *p* = .0021). (H) Expression of miR‐501‐3p in exosomes isolated from control plasmid‐transfected cell media compared with APP plasmid‐transfected cell media (*t*‐test, *p* = .0221) and Tau plasmid‐transfected cell media (*t*‐test, *p* = .0014). (I) Expression of miR‐502‐3p in control plasmid‐transfected cells compared with APP plasmid‐transfected cells (*t*‐test, *p* < .0001) and Tau plasmid‐transfected cells (*t*‐test, *p* = .0105). (J) Expression of miR‐502‐3p in exosomes isolated from control plasmid‐transfected cell media compared with APP plasmid‐transfected cell media (*t*‐test, *p* = .0223) and Tau plasmid‐transfected cell media (*t*‐test, *p* = .0006). Data are presented as mean ± SEM (*n* = 3).

Finally, we aimed to evaluate how the expression of miR‐501‐3p and miR‐502‐3p change intracellularly and if it is secreted extracellularly from the cells in response to AD pathology. First, SH‐SY5Y cells were transfected with control, APP and Tau plasmids. Then, the expressions of APP and Tau proteins were confirmed by immunoblotting and qRT‐PCR analysis. Immunoblotting analysis revealed high levels of both APP and Tau proteins relative to control (Figure 6A). Densitometry analysis of APP and Tau protein showed a significant up‐regulation of APP (*p* < .0001) and Tau (*p* = .0005) in the cells transfected with APP and Tau plasmids, compared with control plasmid‐transfected cells (Figure 6B). Subsequently, a significant fold up‐regulation of APP (∼280‐fold; *p* < .0001) and Tau (∼125‐fold; *p* < .0001) mRNA was observed in the cells transfected with APP and Tau plasmids relative to the control plasmid (Figure 6C).

#### 3.5.2 Characterisation of cell media exosomes

Exosomes were isolated from the media of SH‐SY5Y cells expressing the control, APP and Tau plasmids. The isolated exosomes were characterised by immunoblotting, TEM and DLS analysis. The immunoblotting analysis showed detectable levels of CD63 and TSG101 proteins in all three groups of media exosomes (Figure 6D). Next, TEM analysis showed the cup‐shaped ultrastructure of exosomes in the media from control plasmid, APP‐plasmid and Tau‐plasmid transfected cells (Figure 6E). The exosome vesicle size ranged from 0.102 to 0.210 µm in all three groups—control, APP and Tau cell media exosomes with no significant size differences observed between the groups. Finally, DLS analysis demonstrated exosome size ranging from 10 to 100 nm, with peak measurements consistently around 40 nm across all three cell media exosome groups (Figure 6F). Further, miR‐501‐3p and miR‐502‐3p expressions were analysed inside the cells and media exosomes.

#### 3.5.3 MiR‐501‐3p expression analysis in APP and Tau‐transfected cells and media exosomes

To determine the miR‐501‐3p expression changes intracellularly and extracellularly, qRT‐PCR analysis was performed for miR‐501‐3p on APP‐ and Tau‐expressing cells and their media exosomes. MiR‐501‐3p level was significantly up‐regulated in the cells treated with APP (*p* = .0020) and Tau (*p* = .0021) cells relative to control cells (Figure 6G). Similarly, elevated expression of miR‐501‐3p was detected in the media exosomes of APP (*p* = .0221) and Tau (*p* = .0014) plasmid‐treated cells relative to control plasmid cells (Figure 6H).

#### 3.5.4 MiR‐502‐3p expression analysis in APP and Tau transfected cells and media exosomes

Likewise, intracellular expression of miR‐502‐3p was also significantly elevated in APP‐ (*p* < .0001) and Tau‐ (*p* = .0105) expressing cells relative to control cells (Figure 6I). Further, extracellular expression of miR‐502‐3p was also elevated in the media exosomes of APP‐ (*p* = .022) and Tau‐ (*p* = .0006) expressing cells relative to control media exosomes (Figure 6J). These observations confirm that miR‐501‐3p and miR‐502‐3p expressions change inside the cells, and they are secreted out from the cells in response to both APP and Tau pathology.

### 3.6 | Brain cells study

#### 3.6.1 MiR‐501‐3p and miR‐502‐3p expression analysis in brain cells

**FIGURE 7:**
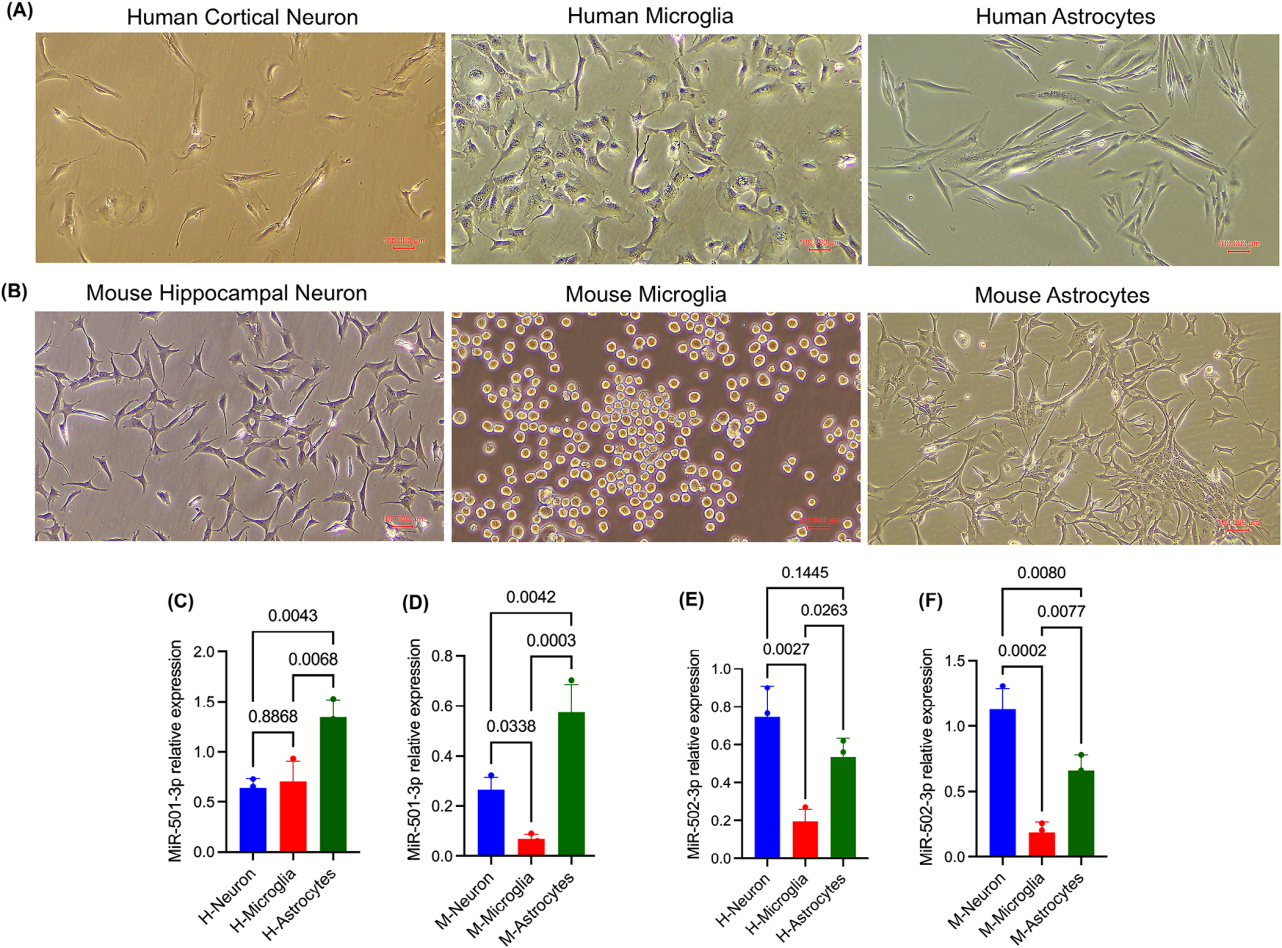
Cell type‐specific expression of miR‐501‐3p and miR‐502‐3p in human and mouse brain‐derived neurons, astrocytes and microglia cells. (A) Representative microscopy images of cortical neurons, microglia and astrocytes derived from human brain (B) Representative microscopy images of hippocampal neurons, microglia and astrocytes derived from mouse brain (40×, scale bars: 100 µm). (C) qRT‐PCR analysis of miR‐501‐3p relative expression in human cortical neurons, microglia and astrocytes (D) qRT‐PCR analysis of miR‐501‐3p relative expression in mouse hippocampal neurons, microglia and astrocytes. (E) qRT‐PCR analysis of miR‐502‐3p relative expression in human cortical neurons, microglia and astrocytes (F) qRT‐PCR analysis of miR‐502‐3p relative expression in mouse hippocampal neurons, microglia and astrocytes. Relative expression of miR‐501‐3p and miR‐502‐3p were calculated using the 2−Δct method. Statistical analysis was performed using one‐way ANOVA with multiple comparisons tests. Data are presented as mean ± SEM (*n* = 3).

To determine the cellular origin of miR‐501‐3p and miR‐502‐3p, the expression levels were examined in neurons, microglia and astrocytes derived from human (Figure 7A) and mouse (Figure 7B) brains. qRT‐PCR analysis revealed relatively high expression of miR‐501‐3p in human astrocytes relative to cortical neurons (*p* = .0043) and microglia (*p* = .0068) (Figure 7C), while in mouse brain, miR‐501‐3p displayed high levels in astrocytes (*p* = .0003) and hippocampal neurons (*p* = .0338) compared with microglial cells (Figure 7D). In contrast, miR‐502‐3p expression was higher in human cortical neurons (*p* = .0027) and astrocytes (*p* = .0263) relative to microglia (Figure 7E) and similarly higher expression of miR‐502‐3p was noted in mouse hippocampal neurons (*p* = .0002) and mouse astrocytes (*p* = .0077) relative to microglial cells (Figure 7F). These results suggest that miR‐501‐3p is predominantly expressed in astrocytes, while miR‐502‐3p is primarily expressed in neurons and astrocytes, indicating these cell types as potential sources of the respective miRNAs.

## 4 DISCUSSION

Diagnostic methods to accurately detect AD remain limited, and similarly, not many treatment modalities currently exist to address AD. With the recent advent of aducanumab, a drug that slows disease progression, there is an increasing need to discover more effective ways to detect AD. In 2011, the National Institute of Neurological and Communicative Disorders and Stroke and the Alzheimer's Disease and Related Disorders Association revised the diagnostic criteria for AD to include Aβ40, Aβ42, Aβ42/Aβ40, t‐tau and p‐tau as CSF biomarkers.^39^, ^40^ However, these biomarkers still struggle to detect the early stages of AD, highlighting the need for continued research to identify new reliable biomarkers. The first established miRNA biomarker was used for diffuse large B‐cell lymphoma in 2008.^41^ Since then, the biomarker potential of miRNAs has been heavily studied in a multitude of diseases, including AD.^42^, ^43^, ^44^, ^45^ While several potential miRNA biomarkers have been identified for AD, none have yet been established for clinical use. With this study, we continue to expand the list of possible miRNA biomarkers and characterise their clinical potential. We have identified increased expressions of miR‐501‐3p and miR‐502‐3p in the AD synapses and have correlated their expression with disease progression based on Braak staging.^33^, ^34^ Moreover, parallel studies in our laboratory are investigating the role of miR‐502‐3p in the modulation of AD proteins, synaptic proteins, mitochondrial morphology and GABAergic synapse function in AD.^35^, ^46^ Therefore, we are interested in evaluating their diagnostic biomarker potential. Many existing studies explored miRNAs in only one sample type (CSF, serum, etc.), which limits our understanding of how miRNAs change in the body during AD pathology. In this study, we analysed miR‐501‐3p and miR‐502‐3p status in AD CSF exosomes, AD serum exosomes, AD fibroblasts, B‐lymphocytes, AD cells, media exosomes and brain cells.

First, we studied the status of miR‐501‐3p and miR‐502‐3p in the AD CSF exosomes. Since CSF is in direct contact with the brain, it is possible that brain cells secrete exosomes enriched with various biomolecules (proteins, miRNAs, metabolites, etc.) in response to AD or neurodegeneration.^47^ Moreover, CSF contains a wide range of biomarkers that can indicate the presence of various neurological diseases.^48^ In AD, for example, CSF levels of Aβ, tau proteins and phosphorylated tau have been shown to correlate with disease stages.^49^ Exosomes contain miRNAs, proteins and other nucleic acids that are packaged within exosomes, which are secreted by various cell types and commonly found in both CSF and serum.^50^ To identify the sources of these exosomes, we performed immunoblotting for cell type‐specific markers. Immunoblotting analysis revealed that most exosomes secreted in the CSF originate from neurons and astrocytes. Our qRT‐PCR results showed a significant increase in the levels of both miR‐501‐3p and miR‐502‐3p in the AD CSF samples compared with UC CSF samples. These findings are consistent with the overexpression of these miRNAs observed in AD synaptosomes.^33^ Since these miRNAs are elevated at the synapse,^33^ and neurons and astrocytes are major players in synapse formation,^51^ it is possible that defective synapses in AD may cause the exosome‐mediated heavy secretion of these miRNAs into the CSF. Moreover, significant AUC values from ROC curve analyses further support their biomarker capabilities. Given the clear differential expression of miR‐501‐3p and miR‐502‐3p, a potential threshold could be established to support AD diagnosis.

Next, we compared miRNA fold change with the neuropathologic reports of the CSF samples to evaluate whether miRNA levels in the CSF exosomes are associated with the severity of amyloid plaques and NFT found in affected brain regions in AD. Upon reviewing the neuropathology reports, we observed that the entorhinal cortex exhibited the highest number of samples with severe amyloid plaques and NFT, followed by the amygdala and hippocampus. Interestingly, most hippocampus or amygdala samples did not show greater severity than the entorhinal cortex areas. This observation suggests that AD pathology progresses in a predictable pattern, with the entorhinal cortex being the first affected brain area in AD.^52^ As mentioned previously, a quantitative analysis of our data is challenging due to the uneven distribution of samples across different severity categories. However, preliminary observations suggest that differential miR‐501‐3p and miR‐502‐3p fold changes could reflect the severity of AD pathology in specific brain regions. To our knowledge, no studies explored the relationship between miRNA fold changes and the neuropathological severity of AD. If such a relationship exists, miR‐501‐3p and miR‐502‐3p could be used as biomarkers of disease progression. This is an area we are particularly interested in investigating further, as such a study could contribute to earlier diagnosis of AD based on miRNA levels.

To further assess the biomarker potential of miR‐501‐3p and miR‐502‐3p, we next evaluated their status in AD, MCI and control serum samples. In the serum, we found that the exosomes could potentially be derived from neurons, astrocytes and microglia. Since serum is more heterogenous than CSF, it could be possible that exosomes may be secreted from a wide range of cells and tissues affected in AD. Our qRT‐PCR results reveal up‐regulation of both miR‐501‐3p and miR‐502‐3p in AD and MCI serum exosomes. The gradual up‐regulation of these miRNAs in control versus MCI versus AD further supports the early AD detection capabilities of these miRNAs. Our findings are well aligned with previous studies of miR‐501‐3p.^53^, ^54^ Previous studies unveiled the elevated levels of miR‐501‐3p on blood exosomes in MCI patients relative to controls^54^ and up‐regulation of miR‐501‐3p in AD brain. However, these studies reported reduced levels of miR‐501‐3p in the serum.^53^ One reason for this discrepancy could be that they studied miR‐501‐3p levels directly from the serum and not the exosomes. Another possibility could be that they used serum samples collected within 2 weeks of the patient's death, while our samples were collected much earlier before their deaths. Exosome biogenesis and subsequent release require energy,^55^, ^56^ and advanced age or severe AD may lead to a decline in ATP levels, potentially affecting exosome production and miRNA levels in the serum.^57^, ^58^ Further studies must be done to explore how serum miRNA levels change with age or with the severity of diseases. Altogether, our findings support the potential of miR‐501‐3p and miR‐502‐3p as serum biomarkers for AD.

Additionally, we investigated the status of miR‐501‐3p and miR‐502‐3p in other peripheral cells, such as B‐lymphocytes and fibroblasts. B‐lymphocytes are one of the major components of peripheral circulation, and their phenotypic and molecular characteristics are altered in Alzheimer's patients,^59^, ^60^, ^61^ which could reflect the changes in miRNA expression. Therefore, we directly assessed the miR‐501‐3p and miR‐502‐3p levels in these cells rather than exosomes. Fibroblasts are not frequent in peripheral circulation; however, their activity is dysfunctional in AD patients,^62^, ^63^ so we focused on investigating the expression of miR‐501‐3p and miR‐502‐3p inside the cells. Our results show that miR‐501‐3p is up‐regulated in fAD fibroblasts; however, there was no significant difference in miR‐501‐3p in sAD fibroblasts. Additionally, there was no significant difference in miR‐501‐3p levels in fAD or sAD B‐lymphocytes. Interestingly, miR‐502‐3p showed an inverse pattern: it was up‐regulated in both fAD and sAD B‐lymphocytes, but no significant difference in miR‐502‐3p expression was observed in fAD or sAD fibroblasts. While further studies are needed to determine whether miRNA levels in fibroblasts or B‐lymphocytes could serve as reliable biomarkers, our results suggest that miR‐501‐3p may be associated with fibroblasts and miR‐502‐3p could be associated with B‐lymphocytes dysfunction during AD pathology.

Further, we investigated how AD pathology might directly influence miR‐501‐3p and miR‐502‐3p levels in intracellular and extracellular secretions. We found that both miRNAs were up‐regulated intracellularly and extracellularly in the cells overexpressing APP and Tau proteins. In APP overexpressed cells, miR‐501‐3p and miR‐502‐3p fold expressions were higher intracellularly than extracellularly. In contrast, Tau overexpressed cells exhibited a higher fold change of miR‐502‐3p in the extracellular media exosomes than intracellularly. This suggests that tau‐related AD pathology may be directly associated with the peripheral secretion of miR‐502‐3p. We also investigated the cellular origin of miR‐501‐3p and miR‐502‐3p in the human brain to understand their potential sources of peripheral secretion in AD. Our data demonstrate that cortical neurons and astrocytes predominantly produce these miRNAs. Specifically, we observed the highest expression levels of miR‐501‐3p in astrocytes and of miR‐502‐3p in both neurons and astrocytes, with significantly lower levels in microglial cells across both human and mouse cells. These findings are consistent with our earlier study reporting elevated levels of these miRNAs in AD synaptic compartments.^33^ Considering that astrocytes support synapse formation and architecture and play a major role in synapse function,^64^ the degeneration of synapses and loss of neurons and astrocytes in AD could potentially explain the secretion of these miRNAs into the CSF and peripheral circulation. This is also supported by a high level of neuronal and astrocyte markers in the CSF exosomes from control and AD samples (Figure 1E), suggesting cellular contributions to extracellular miRNA pools.

One of the primary limitations of our study is the AD specificity and lack of miR‐501‐3p and miR‐502‐3p expression status in non‐AD dementia samples, such as frontotemporal dementia or Lewy body dementia. Unfortunately, we do not have access to other dementia samples. While these miRNAs have been implicated in various diseases within the neurodegenerative spectrum, they have been reported as up‐regulated in vascular dementia and AD.^3^ MiR‐501‐3p and miR‐502‐3p both showed correlation with Aβ and p‐tau tangles in AD brain neuropathology. As such, further research is required to assess the disease specificity of these miRNAs, especially in distinguishing AD from other dementia subtypes. Another limitation is the absence of data correlating miR‐501‐3p and miR‐502‐3p expression with other established AD biomarkers, such as neurofilament light chains or soluble TREM2.^65^, ^66^ Our current cohort lacks information on these markers, and the limited sample volume restricts additional biomarker analyses. Future studies should include a broader panel of AD biomarkers to determine how these miRNAs align with or complement existing diagnostic tools. It is possible that miR‐501‐3p and miR‐502‐3p could serve as part of a multi‐marker panel to enhance diagnostic accuracy and help to differentiate AD from other forms of dementia. However, further validation in larger and more diverse cohorts is essential to explore this potential.

In conclusion, our study thoroughly investigated the biomarker potential of miR‐501‐3p and miR‐502‐3p across different types of AD samples. Altogether, our current findings suggest that miR‐501‐3p and miR‐502‐3p could be a potential biomarker panel and a representation of AD pathology. While we could not confidently define their clinical diagnostic value, the current study has laid a foundation for future research on miR‐501‐3p and miR‐502‐3p and their potential roles in predicting early AD. Further studies with larger sample sizes, including patients with different ethnic groups, are warranted before the preclinical testing of these miRNAs.

## AUTHOR CONTRIBUTIONS

Conceptualisation and supervision: S.K.; D.D. and B.S. contributed equally to this work; experimental performance: D.D., B.S., G.G., D.R., A.K., N.T. and A.P.; analysis, interpretation and validation of data: S.K., D.D., B.S. and A.K.; writing and original draft preparation: D.D., B.S. and S.K.; review, editing and finalisation of manuscript: D.D., B.S., D.R., G.G. and S.K. All authors have read and agreed to the published version of the manuscript.

## CONFLICT OF INTEREST STATEMENT

The author would like to inform that he filed a patent on ‘Synaptosomal miRNAs and Synapse Functions in Alzheimer's Disease’ TTU Ref. No. 2022‐016, U.S. Patent App. No. PCT/US2023/019298 on 26 October 2023 related to the contents of this manuscript. The other authors declare that they have no conflict of interest.

## ETHICS STATEMENT

All experiments were conducted in compliance with the regulation of TTUHSC El Paso and Institutional Biosafety Committe approval for the use of human biological samples obtained from bio banks. Consent was not required as the samples were obtained from the bio banks operated under their respective institution's IRB approvals, with written informed consent from the donors or their families.

## Supporting information



Supporting Information

Supporting Information

Supporting Information

Supporting Information

Supporting Information

Supporting Information

Supporting Information

Supporting Information

Supporting Information

## Data Availability

Data will be made available on request.
