# Supplementary material for: MiRNA‐501‐3p and MiRNA‐502‐3p: A promising biomarker panel for Alzheimer's disease
Source: Clin Transl Med. 2025 Jul 9;15(7):e70389. doi: 10.1002/ctm2.70389 (PMC12238675; doi:10.1002/ctm2.70389)
Supplement: Supplementary file 6 — Supporting Information [file CTM2-15-e70389-s003.docx]

**Supplementary Table 6- Summary of antibody dilutions and conditions used in the immunoblotting analysis**

| **Marker(s)** | **Primary Antibody and Dilution(s)**  **(4^°^C, overnight)** | **Purchased from Company, City & State** | **Secondary Antibody, Dilution(s)**  **(Room temperature, 2 h)** | **Purchased from Company, City & State** |
| --- | --- | --- | --- | --- |
| CD9  (60232-1-Ig) | Mouse monoclonal  1:500 | Proteintech, Rosemont, IL | Rabbit anti-mouse IgG HRP 1:10,000  (A9044-2 mL) | Millipore Sigma  Burlington, MA |
| CD63  (67605-1-Ig) | Mouse monoclonal  1:500 | Proteintech, Rosemont, IL | Rabbit anti-mouse IgG HRP 1:10,000  (A9044-2 mL) | Millipore Sigma  Burlington, MA |
| TSG101  (NB200-112) | Mouse monoclonal  1:500 | Novus Biologicals, Minneapolis, MN | Rabbit anti-mouse IgG HRP 1:10,000  (A9044-2 mL) | Millipore Sigma  Burlington, MA |
| NeuN  (66836-1-Ig) | Mouse monoclonal  1:500 | Proteintech, Rosemont, IL | Rabbit anti-mouse IgG HRP 1:10,000  (A9044-2 mL) | Millipore Sigma  Burlington, MA |
| GFAP  (60190-1-Ig) | Mouse monoclonal  1:1000 | Proteintech, Rosemont, IL | Rabbit anti-mouse IgG HRP 1:10,000  (A9044-2 mL) | Millipore Sigma  Burlington, MA |
| IBA1  (66827-1-Ig) | Mouse monoclonal 1:1000 | Proteintech, Rosemont, IL | Rabbit anti-mouse IgG HRP 1:10,000  (A9044-2 mL) | Millipore Sigma  Burlington, MA |
| APP (6E10)  (NBP2-62566) | Rabbit monoclonal  1:1000 | Novus Biologicals, Minneapolis, MN | Goat anti-rabbit IgG HRP 1:10,000  (A9169-2 mL) | Millipore Sigma  Burlington, MA |
| Tau  (13-6400) | Mouse monoclonal 1:1000 | Thermo Fisher Scientific, MA | Rabbit anti-mouse IgG HRP 1:10,000  (A9044-2 mL) | Millipore Sigma  Burlington, MA |
| GAPDH  (2118) | Rabbit monoclonal  1:3000 | Cell Signaling, MA | Goat anti-rabbit IgG HRP 1:10,000  (A9169-2 mL) | Millipore Sigma  Burlington, MA |
