# Supplementary material for: MiRNA‐501‐3p and MiRNA‐502‐3p: A promising biomarker panel for Alzheimer's disease
Source: Clin Transl Med. 2025 Jul 9;15(7):e70389. doi: 10.1002/ctm2.70389 (PMC12238675; doi:10.1002/ctm2.70389)
Supplement: Supplementary file 7 — Supporting Information [file CTM2-15-e70389-s005.docx]

**Supplementary Table 7. Oligonucleotide sequences of primers used for quantitative reverse transcription-polymerase chain reaction analysis**

| **Gene(s)** | **Sequence(s)** |
| --- | --- |
| Hsa-APP | F- 5^'^ GCCGATGATGACGAGAGAGG 3^'^ |
|  | R- 5^'^ GGGTACTGGCTGCTGTTGTA 3^'^ |
| Hsa-Tau | F- 5^'^ AAAGCCAAGACAGACCACGG 3^'^ |
|  | R- 5^'^ AGCTTCTGCAGGTCGACTCAC 3^'^ |
| GAPDH | F- 5^'^ GCACCGTCAAGGCTGAGAAC 3^'^ |
|  | R- 5^'^ TGGTGAAGACGCCAGTGG 3^'^ |
| U6 SnRNA | F- 5^'^ CGCTTCGGCAGCACATATACTAA 3^'^ |
|  | R- 5^'^ TATGGAACGCTTCACGAATTTGC 3^'^ |
| miR-502-3p | F- 5^'^ AATGCACCTGGGCAAGGATTCA 3^'^ |
| miR-501-3p | F- 5^'^ AATGCACCCGGGCAAGGATTCT 3^'^ |
