# Supplementary material for: MiRNA‐501‐3p and MiRNA‐502‐3p: A promising biomarker panel for Alzheimer's disease
Source: Clin Transl Med. 2025 Jul 9;15(7):e70389. doi: 10.1002/ctm2.70389 (PMC12238675; doi:10.1002/ctm2.70389)
Supplement: Supplementary file 8 — Supporting Information [file CTM2-15-e70389-s001.docx]

**Supplementary Table 8- Details of human and mouse brain cell types.**

| **S. No.** | **Organism** | **Cell type** | **Cat No** | **Designation** | **Source** | **Growth media**  **with 10% FBS 10% and 1x PSN** |
| --- | --- | --- | --- | --- | --- | --- |
| 1 | Homo sapiens | Cortical neuron | CRL-3592 | HCN-2 | ATCC, Manassas, USA | DMEM  (30-2002) |
| 2 |  | Microglial cells | CRL-3304 | HMC3 | ATCC, Manassas, USA | EMEM  (302003) |
| 3 |  | Astrocytic cells | CRL-1718 | CCF-STTG1 | ATCC, Manassas, USA | RPMI  (A1049101) |
| 4 | Mouse | Embryonic hippocampal cells | CLU-198 | mHippoE-14 | Cedarlane labs, North Carolina, USA | DMEM  (11965092) |
| 5 |  | Microglial cells | CRL-3265 | SIM-A9 | ATCC, Manassas, USA | DMEM  (11965092) |
| 6 |  | Astrocyte type 1 clone cells | CRL-2541 | C8-D1A | ATCC, Manassas, USA | DMEM  (11965092) |
