# Supplementary figures and images for: MiRNA‐501‐3p and MiRNA‐502‐3p: A promising biomarker panel for Alzheimer's disease
Source: Clin Transl Med. 2025 Jul 9;15(7):e70389. doi: 10.1002/ctm2.70389 (PMC12238675; doi:10.1002/ctm2.70389)

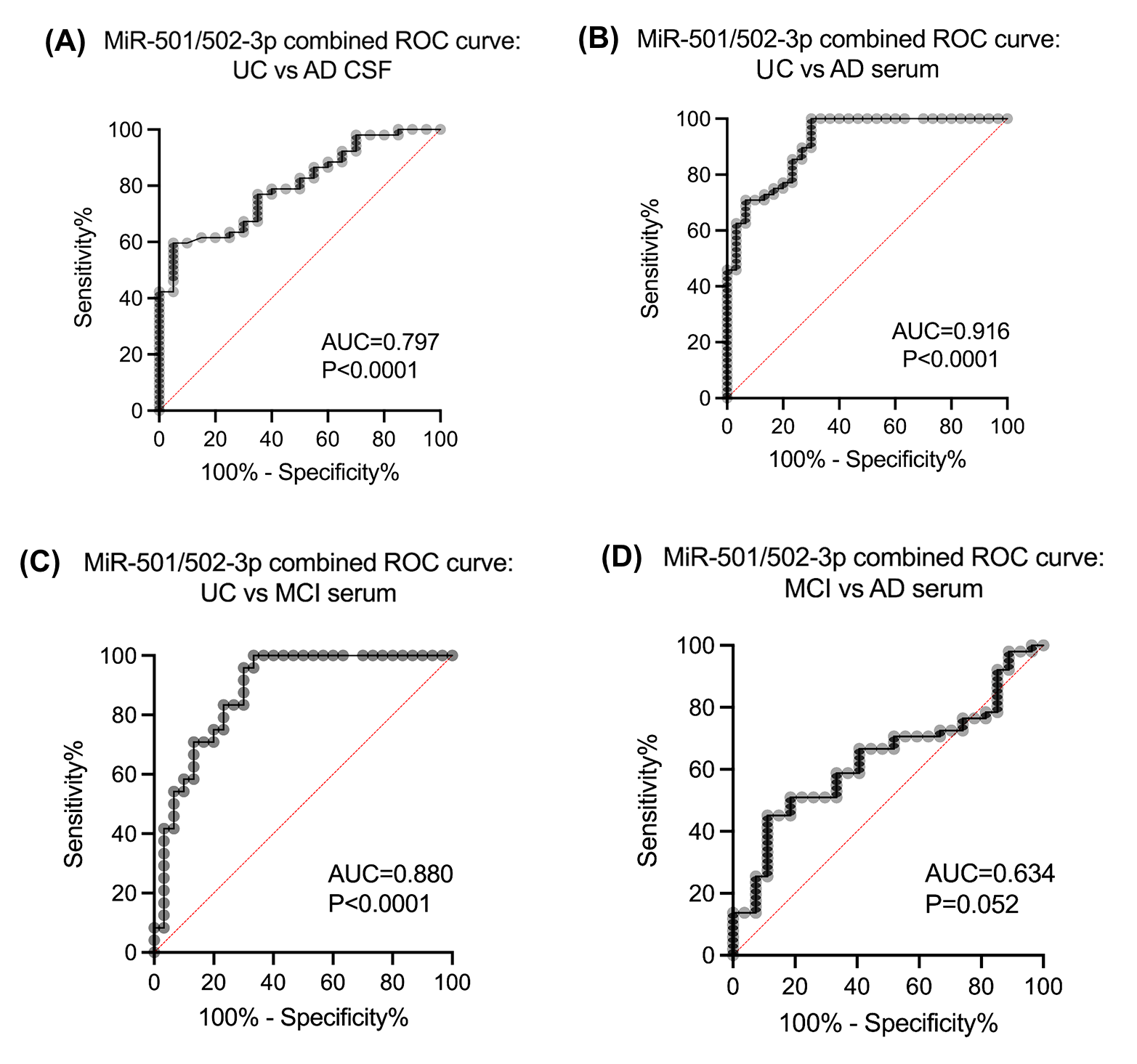

Supplement: Supplementary file 9 — Supporting Information [file CTM2-15-e70389-s004.tif]
